# 物理靶向治疗恶性胸腔积液的机制与临床转化进展

**DOI:** 10.3779/j.issn.1009-3419.2026.101.20

**Published:** 2026-08-20

**Authors:** Jia ZHAO, Zhenyu YIN, Xin HUA

**Affiliations:** 210008 南京，南京大学医学院附属鼓楼医院老年医学科; Department of Geriatrics, Affiliated Nanjing Drum Tower Hospital of Nanjing University Medical School, Nanjing 210008, China

**Keywords:** 恶性胸腔积液, 物理靶向治疗, 压电催化治疗, 声动力治疗, 光动力治疗, Malignant pleural effusion, Physical targeted therapy, Piezocatalytic therapy, Sonodynamic therapy, Photodynamic therapy

## Abstract

恶性胸腔积液（malignant pleural effusion, MPE）是晚期肺癌常见严重并发症，显著影响患者预后。以压电催化、声动力、光动力、胸腔热灌注、射频消融及温度响应水凝胶为代表的物理靶向治疗，通过光、声、压电等能量诱导活性氧生成，或借助局部高温及温控释药继而实现肿瘤杀伤，为MPE治疗提供了新策略。但现有综述多聚焦单一物理技术，缺乏基于胸膜腔特殊微环境及呼吸力学特征的系统分类与适配性比较。本文结合胸膜腔特殊解剖与病理微环境，系统阐述上述技术的作用机制、生物学效应及转化现状，指出多物理场联合、微环境重塑与精准能量调控是未来方向，以期为MPE治疗提供参考。

## Abstract (en)

Malignant pleural effusion (MPE) is a common severe complication of advanced lung cancer and significantly worsens prognosis. Physical targeted therapies, represented by piezocatalytic therapy, sonodynamic therapy, photodynamic therapy, hyperthermic intrathoracic chemotherapy, radiofrequency ablation and thermosensitive in situ hydrogel drug delivery system, induce reactive oxygen species generation via light, ultrasound, or piezocatalytic energy, or achieve tumor killing through local hyperthermia and temperature-controlled drug release, offering new strategies for MPE. However, existing reviews mostly focus on single modalities, lacking systematic classification and suitability comparison based on the pleural microenvironment and respiratory mechanics. This review summarizes their mechanisms, biological effects, and translational status in the context of pleural anatomy and pathological microenvironment, and highlights multi-field combination, microenvironment remodeling, and precise energy regulation as future directions for MPE therapy.

恶性胸腔积液（malignant pleural effusion, MPE）为恶性肿瘤胸膜转移或原发胸膜肿瘤所致的常见并发症，全球年发病率约70/10万，中位生存期为3-12个月，肺癌相关者仅4-6个月^[1]^。其机制涉及血管通透性增加、血管生成增强、淋巴引流受阻，以血管内皮生长因子、炎性因子及免疫细胞交互为核心^[1]^。一线治疗包括胸腔穿刺引流、胸膜固定术、局部化疗及免疫灌注等，但疗效有限^[2]^。胸膜腔作为密闭低灌注腔隙，形成“免疫豁免”微环境，药物受积液再吸收及空间限制难以达到有效浓度^[3]^；同时，患者易并发分隔形成、胸膜增厚粘连及治疗抵抗，进一步限制疗效^[4]^。

近年来，基于物理能量的抗肿瘤策略渐成热点。胸膜腔位置表浅、密闭且呼吸运动规律，有利于物理能量传递与响应，为物理靶向治疗提供了理想场景。其中压电催化治疗（piezocatalytic therapy, PCT）、光动力治疗（photodynamic therapy, PDT）、声动力治疗（sonodynamic therapy, SDT）、胸腔热灌注化疗（hyperthermic intrathoracic chemotherapy, HITHOC）、射频消融（radiofrequency ablation, RFA）及温度敏感型原位水凝胶药物递送系统（thermosensitive *in situ* hydrogel drug delivery system, TSHDDS）等技术通过诱导活性氧（reactive oxygen species, ROS）生成、局部热消融或温控释药实现靶向杀伤肿瘤^[5]^。然而，目前系统总结物理能量靶向治疗MPE的综述仍较少。本文就其机制、现状及临床转化进行综述，以期为临床决策提供参考。

## 1 胸膜腔特殊构造与微环境在物理靶向治疗中的优势

### 1.1 胸膜腔的解剖生理特点与物理靶向治疗的适配性

胸膜腔具有独特的解剖与生理学特点，使其在物理靶向治疗中优势突出：作为封闭腔隙，其物理能量不易损耗^[6]^；积液的流动性利于纳米敏化剂均匀分散，提高病灶覆盖率^[7]^；胸膜肿瘤多表浅弥漫或结节状浸润，便于能量精准作用^[8]^；呼吸压力波动可驱动压电材料产生ROS，实现无需外接电源的自主抗瘤^[9]^。

基于上述解剖生理特点，6类技术对胸膜腔的适配性各有侧重：HITHOC借助胸腔液体实现全腔隙覆盖；PDT与RFA依赖光能或局部能量沉积，适用于局限性病灶；SDT利用超声增强深部能量递送；TSHDDS实现药物原位滞留与缓释；PCT利用呼吸压力持续产生ROS。由此可从空间封闭性、液体环境、能量可达性及作用范围构建“胸膜腔专属物理治疗适配性评价体系”，为技术定位与联合应用提供依据。

### 1.2 胸膜腔的病理微环境对物理治疗的协同增效作用

肿瘤快速增殖与糖酵解形成缺氧、酸性及高氧化应激微环境，既促进MPE进展，也为物理治疗提供了窗口^[10]^。PCT、SDT及PDT可借异常氧化还原状态增强ROS介导的抗肿瘤效应^[11]^；RFA和HITHOC通过局部热效应直接杀伤并提高治疗敏感性^[12,13]^；TSHDDS依托封闭的胸膜腔实现持续精准释药^[14]^；物理治疗还可诱导免疫原性细胞死亡（immunogenic cell death, ICD），逆转免疫抑制^[8]^。因此，该微环境有利于局部消融、持续释药、微环境重塑及免疫激活。

## 2 物理靶向治疗MPE的核心机制

物理靶向治疗MPE的核心在于利用可控能量精准干预胸腔内肿瘤细胞及微环境，实现抗肿瘤及抑制积液生成，其主要机制可归纳为以下几个方面。

### 2.1 物理能量介导的肿瘤细胞直接杀伤机制

物理靶向治疗主要通过两类机制直接损伤肿瘤：一是产ROS类，如PDT以特定波长激光激活光敏剂，产生单线态氧与自由基^[15]^；SDT经超声激活声敏剂产生ROS^[16]^；PCT借呼吸运动诱导电荷分离并生成ROS，实现持续氧化杀伤^[17]^。二是热/温控类，RFA将交变电能转为局部热能以致蛋白变性及凝固坏死^[18]^；HITHOC以外源温热直接损伤肿瘤^[19]^；TSHDDS利用生理热能触发溶胶-凝胶相变，原位形成药物储库，实现长效缓释^[20]^。

### 2.2 胸膜纤维化调控与积液生成抑制机制

MPE的发生与胸膜肿瘤侵犯所致毛细血管通透性增加及淋巴回流受阻密切相关^[21]^，而物理靶向治疗通过调控胸膜局部组织状态减少积液形成。例如，RFA或HITHOC可对胸膜表面肿瘤结节产生热损伤，诱导间皮细胞发生可控炎症反应与纤维化，促进胸膜腔闭合，减少液体渗出^[22,23]^。此外，物理能量还可抑制肿瘤细胞分泌促血管生成因子，降低胸膜微血管通透性与异常血管生成，进一步减少积液产生^[24]^。

### 2.3 物理能量介导的靶向递送与协同增效机制

物理磁场可增强治疗制剂在胸腔肿瘤部位的富集，实现精准递送并提升疗效。例如，磁性纳米药物可在体外磁场引导下靶向富集于胸膜转移灶，实现局部高浓度释放并降低系统毒性^[25]^。此外，超声介导的空化效应通过微泡振荡与瞬态孔隙直接破坏肿瘤细胞膜结构，同时增加胸膜组织通透性，促进药物及免疫细胞向肿瘤组织渗透，显著增强治疗效果^[26]^。

### 2.4 免疫微环境激活与协同抗肿瘤机制

物理靶向治疗还可通过调控胸腔局部免疫微环境发挥协同抗肿瘤作用。如肿瘤细胞经物理能量损伤后释放大量肿瘤相关抗原及损伤相关分子模式，促进树突状细胞（dendritic cells, DCs）摄取与呈递，激活适应性免疫^[27]^。此外，部分物理治疗可减少调节性T细胞（regulatory T cells, Tregs）和髓源性抑制细胞（myeloid-derived suppressor cells, MDSCs）比例，促进CD8^+ ^T细胞浸润与活化，形成持续抗肿瘤免疫应答，降低胸膜转移与积液复发风险^[28]^。

### 2.5 多物理场联合治疗协同机制

单一物理治疗常受限于能量穿透、氧供、敏化剂滞留或作用时间，多物理场联合可通过能量互补放大疗效。SDT联合PDT以超声深层穿透弥补PDT光穿透不足，双功能敏化剂在声/光激发下协同产ROS，诱导ICD，促进DCs成熟与CD8^+ ^T细胞浸润，并降低Tregs及MDSCs比例以逆转免疫抑制^[29-31]^。PCT联合HITHOC或智能递药系统的协同则侧重时空互补：PCT经呼吸驱动压电效应持续产ROS并诱导ICD^[32]^；HITHOC通过热效应增强胸膜通透性与药物转运^[33]^；温敏水凝胶原位成胶形成储库，延长局部滞留并抑制血管生成^[14]^。联合策略实现“持续供能→热增敏促转运→缓释延长暴露”的级联协同，弥补单次物理治疗作用时间有限的不足。

综上，多物理场联合并非简单叠加，而是通过能量互补、ROS放大、ICD增强及持续递药，协同突破单一治疗局限，推动胸膜腔由免疫抑制向免疫激活转变。

## 3 目前胸腔内物理靶向治疗的主要方式

依据作用机制，胸腔内物理靶向治疗可分为产ROS类（PCT/SDT/PDT）、热消融类（RFA/HITHOC）及智能递药类（TSHDDS），分别通过诱导ROS生成、局部热效应及温度响应控释实现抗肿瘤作用。

### 3.1 产ROS类物理靶向治疗方式

#### 3.1.1 PCT：基于呼吸驱动的自供能抗肿瘤策略

PCT是一种机械能驱动的原位催化技术：呼吸运动引起胸腔周期性形变，使压电材料电荷分离，将内源机械能转化为电能，催化氧或水分子持续产生ROS，实现无创、自供能抗肿瘤并调控微环境^[17]^。前期研究^[9]^显示，胸腔内压力驱动的压电纳米酶可抑制胸膜肿瘤增殖、减少积液，并对耐药肿瘤有效。近期报道的单原子掺杂压电催化剂在机械刺激下产生ROS，激活非铜依赖性铜死亡，同时触发铁死亡与铁蛋白自噬，协同增强肿瘤抑制^[34]^，也为难治性MPE提供了新策略。

#### 3.1.2 SDT：基于组织穿透与空间可控靶向的无创胸腔物理治疗策略

SDT是一种非侵入性技术，利用低强度超声激活声敏剂，凭借组织穿透力适用于深部肿瘤^[35]^。其核心机制为超声空化效应：气泡振荡塌陷产生瞬时高温高压，促进自由基生成并提高膜通透性，利于声敏剂与氧富集，进而大量产生ROS，引发脂质过氧化及DNA损伤，破坏线粒体膜电位并激活凋亡通路，诱导肿瘤细胞死亡^[26]^。同时，SDT可触发ICD并激活全身抗肿瘤免疫^[36]^；多功能纳米声敏剂通过增强靶向富集进一步强化这一协同免疫效应，形成联合治疗模式^[37]^。

#### 3.1.3 PDT：基于直视引导的胸膜肿瘤精准物理治疗策略

PDT利用光敏剂、特定波长光与氧分子，局部照射后经I/II型光化学反应产生ROS，损伤亚细胞器并诱导细胞死亡^[38]^。在MPE治疗中，PDT多经胸腔镜直视照射，精准处理胸膜结节，并联合胸膜固定术处理分隔粘连^[24]^；手术减瘤后PDT可覆盖脏层胸膜病变及残留的不可见癌灶，通过光化学损伤、血管破坏及免疫激活实现区域控制^[24]^。但PDT受组织穿透有限、氧依赖及皮肤光毒性限制，单独应用受限，与SDT联合可协同增效^[37]^。

### 3.2 热消融类物理靶向治疗方式

#### 3.2.1 RFA：基于高频电热效应的精准局部消融策略

RFA经电极针传递高频交变电流，使组织内离子振荡产热，形成60-100 ^o^C高温区，致蛋白凝固坏死^[12]^；还可诱导热应激分子释放，促进肿瘤抗原暴露，激活DCs及T细胞介导的抗肿瘤免疫^[39]^。对胸膜转移或肺内病灶，RFA可减少促血管通透性因子释放，抑制积液生成^[40]^，有望成为MPE综合治疗的重要补充，为不宜手术者提供局部治疗选择。

#### 3.2.2 HITHOC：基于局部高温增敏与胸腔广泛覆盖的区域治疗策略

HITHOC将局部热疗与胸腔灌注化疗结合，利用41-43 ^o^C高温促进药物渗透，诱导蛋白变性及DNA损伤，并增加细胞膜流动性与血管通透性，提高局部药物浓度，实现热化协同增效^[19,41]^。针对MPE弥漫性分布，HITHOC通过循环灌注使加热化疗药物均匀覆盖胸膜腔，作用于微小转移灶及游离细胞，提高局部药物浓度并减少全身不良反应^[19]^。因此，HITHOC是恶性胸膜播散及MPE局部控制的重要区域治疗策略。

### 3.3 智能递药类物理靶向治疗方式——TSHDDS：基于原位成胶与持续控释的局部递药策略

TSHDDS是一种体温触发溶胶-凝胶转变的智能递药系统，温敏聚合物形成三维网络，将药物固定后持续释放，延长局部暴露、提高疗效并降低全身毒性^[42]^。针对传统灌注药物易被快速清除的局限，其可在原位形成药物储库，实现稳定释药并延长局部滞留^[43]^；同时可负载化疗药、纳米酶及声敏剂等，构建多模式联合平台^[44]^。在MPE研究中，Esculentoside A负载的温敏PLEL水凝胶胸腔注射后可持续释药，显著抑制MPE进展并延长动物生存，其机制与减少巨噬细胞浸润及血管生成有关^[20]^；负载胸膜固定剂亦可提高覆盖范围与滞留时间，改善固定效果^[45]^。

## 4 物理靶向治疗MPE的临床转化现状、优势获益人群及面临的挑战

### 4.1 各模态物理靶向治疗的临床转化进展

PCT及TSHDDS仍处于临床前阶段，研究重点集中于材料生物相容性优化与胸腔内长期滞留，两者分别通过呼吸驱动自供能ROS生成及原位药物储库延长暴露时间，展现出MPE控制潜力，但尚缺乏人体临床数据。SDT在MPE专项临床研究尚属空白，仅在其他实体瘤中有少量个案级临床探索^[46]^。PDT是临床证据最充分的技术之一，多项I/II期及随机对照研究（randomized controlled trial, RCT）^[24,47-50]^证实其安全性与可行性。HITHOC已有临床研究及多项*meta*分析支持其提高积液控制率并改善生存获益^[41,51-53]^。RFA在MPE中的直接证据有限，现有研究^[40,54]^多针对局限性病灶或与胸腔灌注联合应用，随着影像导航及精准消融技术发展，有望成为综合治疗的重要补充。可见不同物理治疗模式的临床转化进程差异显著，见表1^[24,40,47-50,52,54]^。

**表1:** MPE相关物理靶向治疗的临床转化研究进展

Authors	Year	Study type	Therapy	Tumor type	Sample size	Main results	Adverse events	Median effusion recurrence-free time	Follow-up duration
Mai Z et al.^[24]^	2025	Pilot study	Thoracoscopic PDT±APC	MPE (mainly NSCLC)	28	12-week OS rate: 89.3%; 1-year OS rate: 75%	No severe AEs; mild chest pain/fever, well tolerated	NR	NR
Friedberg JS et al.^[47]^	2004	Phase II	Surgery+PDT	Lung cancer with pleural spread	22	Local control rate: 73%; OS: 21.7 mon	Perioperative complications	NR	NR
Pass HI et al.^[48]^	1997	Phase III	Surgery+PDT	Malignant pleural mesothelioma	63	No OS improvement	Surgery-related complications	NR	NR
Takita H et al.^[49]^	1994	Phase II	Surgery+intracavitary PDT	Malignant pleural mesothelioma	23	OS: 12 mon	>50% postoperative complications; 2 deaths	NR	NR
Rice SR et al.^[50]^	2019	Prospective Phase II	PDT+proton therapy	Advanced mesothelioma	10	2-year local control rate: 90%	AF, pneumonia, DVT; no ≥ grade 2 AEs	NR	>2 years
Li ZH et al.^[52]^	2023	Retrospective	HITHOC	Lung cancer with MPE	33	3-month MPE control rate: 69.6%; prolonged OS	NR	22.4 mon	NR
Shen BR et al.^[40]^	2021	RCT	RFA+thoracic targeted perfusion	Lung cancer with MPE	101	Combined group prolonged MPE PFS (P<0.05)	No grade 3/4 severe AEs	NR	NR
Kodama H et al.^[54]^	2026	Retrospective	RFA	Recurrent pleural mesothelioma	14	Local control rate: 92%; 1-/3-/5-year OS rate: 100%/100%/60%	Major complications: 4.3%; minor complications: 4.3%	NR	NR (5-year OS estimated)

MPE: malignant pleural effusion; NSCLC: non-small cell lung cancer; PDT: photodynamic therapy; APC: argon plasma coagulation; HITHOC: hyperthermic intrathoracic chemotherapy; RFA:radiofrequency ablation; OS: overall survival; PFS: progression-free survival; RCT: randomized controlled trial; AEs: adverse events; AF: atrial fibrillation; DVT: deep vein thrombosis; NR: not reported.

从循证医学层面看，各技术呈现“临床前-早期转化-临床应用”三级成熟度梯度，见表2^[8,40,41,43,46,48,49,51-54]^。PCT与TSHDDS均缺乏人体I期数据；SDT仅少量胸腔探索性试验；PDT的II期研究显示治疗潜力，但III期RCT未证实生存获益，证据等级中等偏低；HITHOC已纳入国内专家共识，但原始研究异质性较高，仍需多中心RCT明确最佳参数与适宜人群；RFA囿于仅适用于局限性病灶的临床局限。

**表2:** MPE 物理治疗技术分层对比

Therapy	Translation stage	Core evidence level	Suitable lesion type	Current clinical positioning
PCT	Preclinical	Animal studies^[8]^	Potentially for diffuse MPE	No phase I evidence
TSHDDS	Preclinical	Animal/material studies^[43]^	Diffuse pleural surface lesions	No phase I evidence
SDT	Early translation	Case report (breast cancer with pleural metastasis)^[46]^	Deep/local pleural lesions	No MPE-specific evidence; early feasibility stage
PDT	Clinical application	RCTs (phase III, no survival benefit)^[48]^+ phase II^[49]^	Pleural dissemination, superficial lesions	Exploratory basis; survival benefit unconfirmed
HITHOC	Clinical application	Multiple meta-analyses^[41,51,53]^+retrospective studies^[52]^	Lung cancer with MPE (diffuse)	Mainstay of intrapleural regional therapy
RFA	Clinical application	RCTs^[40]^+retrospective studies^[54]^	Limited lesions only; not for diffuse metastasis	Common technique for local precision therapy

PCT: piezocatalytic therapy; TSHDDS: thermosensitive in situ hydrogel drug delivery system; SDT: sonodynamic therapy.

### 4.2 物理靶向治疗的潜在获益人群

综合现有证据，物理靶向治疗在以下人群中更具优势：（1）耐药MPE患者：通过ROS发挥非靶点依赖的抗肿瘤作用，联合局部缓释或免疫治疗可增效；（2）包裹性MPE：物理能量可松解粘连、破坏纤维分隔屏障，联合胸腔镜处理分隔病灶，改善局部疗效；（3）不耐受常规治疗者：SDT、PDT等局部治疗全身毒性低，耐受性更优；（4）晚期姑息患者：长期控液、减少穿刺，符合姑息治疗的原则。

在此基础上，可进一步结合胸水多组学标志物、胸膜病灶影像学特征及2023版中国专家共识^[2]^，建立个体化分层匹配框架：依据患者体力状态评分、肿瘤类型及实验室指标进行预后评估，并结合排液后肺复张状态制定局部策略。具体而言，包裹性积液或局限性可定位病灶优先评估胸腔镜下PDT或RFA；弥漫性胸膜播散评估HITHOC的适用性；耐药、晚期或体弱难耐受侵入性治疗者探索无创SDT；胸膜腔内局部给药则可利用温敏水凝胶或压电纳米材料的长期滞留特性，实现微环境响应的缓释治疗。上述路径仍需前瞻性临床研究验证。

### 4.3 面临的问题与挑战

当前胸腔物理靶向治疗仍面临多重挑战：如多种物理功能材料（如光敏剂、声敏剂、压电材料及磁性纳米载体）尚处早期，其长期安全性、降解代谢及靶向富集效率等关键问题亟待研究^[55]^；治疗参数（能量、范围、穿透深度、时间及频次）缺乏统一规范，剂量-效应及安全阈值待明确；临床证据多为小样本探索性研究，缺乏RCT及长期随访验证^[24]^；物理与系统/免疫联合治疗的协同机制及最优策略也有待进一步探索。

## 5 未来发展方向

未来MPE物理靶向治疗将向精准化、协同化与个体化发展。多模态联合（PCT、SDT、PDT、RFA、HITHOC及TSHDDS）有望协同增效；人工智能辅助影像导航与实时能量监测可提升精准性与安全性；基于胸水多组学的分层体系有助于筛选优势人群。在此基础上，材料与设备升级是实现上述目标的关键支撑。新型智能响应材料将持续推动胸腔局部治疗平台升级，温敏水凝胶、压电纳米材料及多功能声敏/光敏纳米载体可实现长效滞留与精准释药，提高局部疗效并降低全身毒性。结合我国临床资源与材料研发基础，未来可重点推进可降解压电纳米酶、国产温敏水凝胶制剂及便携式胸腔超声声动力设备等低成本、高可行性本土转化，并围绕安全性、胸腔滞留时间、能量参数及剂量标准化建立系统评价体系，加速实验室向临床转化。

与此同时，物理治疗的长远价值不仅在于局部控瘤，更在于免疫微环境重塑与长期管理。物理联合免疫治疗可通过诱导ICD重塑胸膜腔微环境，将物理杀伤的即刻效应转化为持续免疫监视，为长期疾病控制奠定基础。长远而言，物理治疗不应局限于短期控液，而应致力于胸膜微环境稳态调控与远期复发率降低。未来可将积液控制率、无复发时间、胸膜病灶、症状及生活质量纳入长期随访体系，并依据患者预后分层，将物理治疗与全身治疗、胸腔引流及姑息支持治疗有机协同，推动其融入MPE全程多学科管理体系。

## 6 小结

MPE为晚期恶性肿瘤常见并发症，病理特征为血管生成异常、免疫抑制、肿瘤播散及药物分布受限。当前以引流、胸膜固定及全身治疗为主，但疗效有限。近年PCT、SDT、PDT、RFA、HITHOC及TSHDDS等物理策略通过ROS、热效应、消融及缓释机制，精准干预胸膜病灶与微环境，具有抑瘤、控液及免疫调控潜力。随着材料、能量调控及转化医学的发展，物理靶向治疗有望成为MPE综合治疗的重要组成，推动其由姑息控制向精准干预与长期管理转变。
